# Quality assessment of fish vaccine data in the Norwegian Veterinary Prescription Register (VetReg)

**DOI:** 10.1186/s12917-024-04460-7

**Published:** 2025-01-13

**Authors:** Trishang Udhwani, Katharine R. Dean, Ingunn Sommerset, Kari Olli Helgesen

**Affiliations:** Norwegian Veterinary Institute, Elizabeth Stephansens Vei 1, Ås, 1433 Norway

**Keywords:** Aquaculture, Vaccination, Data validation, Animal health data, Salmon, Biosecurity, Secondary data

## Abstract

**Background:**

Vaccination of farmed salmonids has been an integral part of preventing infectious diseases in Norway’s aquaculture industry. In Norway, vaccine usage is regulated by the government. There is a need to monitor vaccine usage for both regulatory and research purposes, at local and national scales. The Norwegian Veterinary Prescription Register (VetReg) is a national database that includes all prescriptions of medicines to animals dispensed by pharmacies and all medicines used for food producing animals by veterinarians. This study aimed to evaluate the quality of fish vaccination data reported to VetReg in 2016–2022. We considered the following attributes: completeness, validity, and timeliness. For external validation, we compared the data in VetReg to wholesaler statistics.

**Results:**

Pharmacies reported fish vaccines to VetReg in a variety of quantity units, including doses and volumes, which required us to harmonize the data to a single unit. It was not possible to harmonize the quantity units for nine percent of the records, which were mainly bath vaccines reported in doses. We identified specific issues that required manual editing of the units of 1 percent of the records. We validated individual variables such as product codes and location identifiers using external registers. The ‘number of animals’ variable was inconsistent for 31 percent of the records. The coverage of vaccine data in VetReg ranged from 81 to 113 percent for the ten most sold vaccines in 2020–2022, as compared to wholesales statistics. For the timeliness, we found that 75 percent of the records were submitted within 25 days for all years.

**Conclusions:**

Overall, we found that the fish vaccination data in VetReg was of sufficient quality to monitor injectable vaccine usage at hatcheries after 2020. We identified issues at the product level, with bath vaccines, and with single variables (number of animals, weight, and species). We recommend that quality can be improved by reporting all vaccines in volume rather than doses, reporting a single vaccine prescription per report, and including a deadline for pharmacies to report in the legislation.

**Supplementary Information:**

The online version contains supplementary material available at 10.1186/s12917-024-04460-7.

## Background

In Norway, aquaculture production of Atlantic salmon and rainbow trout is an economically important industry that continues to expand. Norwegian aquaculture is primarily focused on the cultivation of these two species. Norway is currently the world’s largest producer of Atlantic salmon with a production of 1.55 million tons in 2022 and an export of 1.2 million tons in 2023 [1, 2]. The intensification of salmonid farming would not have been possible without effective disease control and prevention measures. In the late 1980s and early 1990s, vaccination contributed significantly to the growth of salmonid aquaculture, as well as, the low antibiotic usage seen for many years in Norwegian fish farming, by preventing important bacterial diseases [3, 4]. However, diseases caused by parasites, bacteria and viruses, continue to threaten the fish health and welfare of Atlantic salmon and rainbow trout [5].

The production of Atlantic salmon and rainbow trout mimics their natural history as anadromous fish. Farmed salmonids begin their lives in freshwater hatcheries, where they are kept until they have smoltified. After smoltification, the fish are physiologically adapted to a life in seawater. The smolts are then transferred to seawater facilities where they are kept until they are slaughtered. In 2022, hatcheries sold 418 million Atlantic salmon and 26 million rainbow trout to on-growing sea farms [6]. Nearly all of these fish were vaccinated against one or more pathogens, which takes place at the hatcheries [5]. Today, there are several vaccines available to protect salmon against major bacterial and viral pathogens. However, effective vaccines against parastic diseases are limited.

The law regulates the use of vaccines for farmed salmonids in Norway. Vaccines are prescribed by veterinarians. At a basic level, all medicines (including vaccines) prescribed for fish must have marketing authorization in Norway or be granted a special license from the Norwegian Medical Product Agency (NoMA). Vaccines for fish additionally require permission for use by the national competent authority on fish health and welfare, the Norwegian Food Safety Authority (NFSA) [7]. The regulation of vaccines covers both the use of certain vaccines and the prohibition of others. Presently, the NFSA states that farmers should vaccinate Atlantic salmon and rainbow trout against vibriosis, furunculoses, and cold-water vibriosis [8]. On the contrary, there are fish diseases where early detection and eradication is important [7]. To prevent immunity from vaccination interfering with early disease detection, the government may choose to prohibit vaccination against certain diseases. Similar regulations can be applied to specific geographic areas or compartments when there is a goal to obtain “disease-free” status. Disease-free status can be important for gaining access to specific markets internationally due to import restrictions.

There is a need to monitor vaccine usage for several reasons. Firstly, it is important to know the vaccination status of the farmed fish in order to evaluate to what extent the farmers follow the legislation. Secondly, the vaccination status of the fish is an important factor to understand the underlying reasons for the prevalence and spread of infectious diseases. For example, knowledge of vaccination status is required to understand how well a vaccine protects against a certain disease and to compare the efficacies of different vaccines for the same disease [9]. Finally, some vaccines may cause harmful side effects to the fish and therefore it is important to monitor their usage to protect fish welfare [10]. For example, the vaccination status is needed in order to evaluate side effects of specific vaccines and combinations of vaccines. Studies of vaccine side effects are mainly conducted by pharmaceutical companies during clinical and field trials. They have access to vaccine status of the fish through company records. If others wanted to evaluate side effects, the veterinary prescription registry (VetReg) could be a potential source of the vaccination status.

Information about vaccine usage in salmonids in Norway can be obtained from multiple sources including wholesaler statistics, the national prescription register, and farm records. Sales can be followed at a national level through the wholesalers' statistics reported annually to the Norwegian Institute of Public Health (NIPH), where wholesalers of medicines are obliged to enter their medicine sales [11]. Vaccine sales can be obtained from VetReg owned by the NFSA [12]. Pharmacies are obliged to report all sales of prescribed medicines for all animals and medicines sold directly to veterinarians. For veterinarians and authorized fish health personnel it is mandatory to report the use of medicines for food-producing animals, including fish. Reports to VetReg are per prescription or delivery to veterinarians (for pharmacies) or per treatment of an animal or group of animals for veterinarians. Reporting to VetReg has been mandatory for medicines used for fish since 2011. Finally, records at hatcheries also contain detailed information about the vaccine status of each fish group, although these may be harder to obtain at a national level.

The national statistics generally do not have information about autogenous vaccines, which are vaccines produced for a specific farm against a variant of a pathogen found in the same farm. It is legal to use these vaccines, but only if there are no commercial vaccines with marketing authorization available against the pathogen or the available vaccines have low or insufficient efficacy. It is difficult to follow the vaccine status of autogenous vaccines at any level without access to company records because these vaccines are not authorized in any country. This means that there are no publicly available data for the active substances, dosage regime, route of administration, and target species.

VetReg can be an appropriate source to provide timely and accurate information about vaccinations used in Norwegian aquaculture for Atlantic salmon and rainbow trout. The quality of the register has been assessed previously for antibiotic use data and for data on anti-parasitics used against salmon lice [4, 13, 14]. However, there are no previous studies performing quality assessment or validation of fish vaccination data in VetReg. Birkegård et al. [15] proposed a framework for assessing the data quality of nationwide animal health registers, including a description of the register, use of relevant quality attributes, and identification and communication of quality issues [15]. Here we use this framework to evaluate VetReg for fish vaccination data in Norway from 2016–2022. Based on our evaluation, we make recommendations for using and improving the VetReg data for fish vaccines.

## Methods

### VetReg

VetReg covers all prescriptions of medicines for animals dispensed to animal owners from pharmacies, including all medicines sold from pharmacies to veterinarians. In addition, it covers all prescription medicines used for food-producing animals (including horses) and voluntary reports for animals kept for other purposes, reported by veterinarians [12]. The deadline for reporting is seven days for veterinarians, while it is to the best of our knowledge not stated for pharmacies. In general, access to the register data is restricted and we obtained the data through an existing agreement between the Norwegian Veterinary Institute and the NFSA.

We received the VetReg data as a comma separated (.csv) file, extracted by the NFSA from their centralized database on 17 Jan 2024. The extracted data included variables originating from VetReg (report ID and date of registration), from the national product registry (Anatomical Therapeutic Chemical (ATC) code, national product ID, product name, pack size, pack size units), and from pharmacies or veterinarians (quantity, quantity units, animal species, number of animals, weight of the animals, date of delivery, name of pharmacy and animal health professional, information about the owner of the animal(s) (only for food producing animals), identification number of the animal(s), reason for prescription and diagnosis).

From the complete dataset, we filtered the data based on the date of delivery for the study period for each individual year from 2016–2022 and ATC codes associated with fish vaccines for salmon or rainbow trout, QI10A and QI10B. Our study was limited to the period from 2016 to 2022, as these were the years for which we had access to both VetReg and wholesaler data. We did not find any records of autogenous vaccines in VetReg. If autogenous vaccines were reported to VetReg, the records would not be included when filtering for ATC-codes, since these codes come from the national product registry where autogenous vaccines are not registered.

Each row of the data represented a registration made by a pharmacy or veterinarian/animal health professional. For fish vaccines, we expected that all registrations to VetReg to be marked as “Delivery from pharmacies to animal owners/husbandry”. However, there were records marked as “Notification of animal health professional’s use of medicinal products” in all years of the study period except in 2017 and 2020. According to the regulations, animal health personnel should only report the use of medicines first dispensed to themselves by a pharmacy [12]. Upon investigation, we did not find any records of animal health personnel buying any fish vaccines from pharmacies except a single record in 2018. The relevant veterinarian had not reported the use of these vaccines. Moreover, veterinarians who reported the use of vaccines were the prescribers of vaccines for same fish farms during the same period. Therefore, we removed all entries marked “Notification of animal health professional’s use of medicinal products” as likely duplicates to the dispensations already reported by pharmacies. Further data cleansing of the VetReg data is described in the validity section.

### Wholesaler data

Wholesaler data are nationwide data on medicines sold from wholesalers to Norwegian pharmacies. These data are collected by the Norwegian Institute of Public Health (NIPH) and are mandatory to report according to regulation [11]. The deadline for this report is 15th of January the following year. We asked the NIPH for wholesaler data for ATC code QI (vaccines for animals including fish) for 2016 to 2022. We received the wholesale data on 3 Jan 2024 as an excel file. The data we received contained information with the year, ATC-code, national product ID, product name, pack size, number of packages sold per year and whether or not the product was marketed in Norway or imported via special license. We filtered the data for ATC-codes QI10A and QI10B. We excluded entries for the vaccine “IZOVAC ENCEPHALOMYELITS” due to incorrect ATC coding. For this vaccine, the correct ATC-code is QI01AD02 [16]. We further excluded data for autogenous vaccines (two vaccines reported in 2016), since no records of the use of autogenous vaccines was found in our VetReg dataset.

### Validity

In the context of data quality assessment, validity refers to “whether or not the register includes the true value” [15]. For VetReg, we evaluated the internal and external validity as appropriate for different variables in the dataset.

We checked the reported quantities and quantity units (e.g. dose, ml) for unexpected values. We simultaneously performed data cleansing to prepare the data for completeness evaluations by converting reports for injectable vaccines in doses to ml using the information given for each national product ID in the Summary of Product Characteristics (SPC) found using the medicine database “legemiddelsøk” administrated by the Norwegian Medicinal Products Agency (NoMA) [17]. The goal was to get a common unit for all use to be able to compare with wholesales data. Records reported in ‘g’ and ‘kg’ were considered invalid. However, we tried to identify if the if the actual reporting unit was the corresponding volume unit (‘ml’ and ‘L’, respectively) or ‘stk’ (meaning dose). This was done by using “number of animals” column as a reference. For records reported in “g” or “kg”, we calculated the number of doses by assuming the reported unit “ml” or “L”, respectively. If the number of doses calculated was equal to the number of animals, we considered the assumption to be true and we manually corrected the unit. Otherwise we flagged the records for validity errors. These records were not included in the completeness evaluation because of incorrect units. For bath vaccines, the number of doses given from each preparation can vary, thus it is not possible to convert number of doses to ml. Bath vaccine records with the reporting unit “dose” were therefore excluded from further analysis. To address if the units reported as doses should have been ml, and vice versa, we investigated if the number of animals exactly matched the number of doses calculated from quantity given in ml (doses incorrectly coded as ml) or the number of animals exactly matched the number of doses (ml incorrectly coded as doses). These records were corrected as a data cleansing step prior to the completeness evaluation.

We checked that the reported species was reasonable. Furthermore, we also checked what year the vaccine was authorized for use in Norway, although vaccines can also be imported from another county where they are authorized, via special license from NoMA. All aquaculture sites in Norway are identified by a 5-digit location number that can be found in the Norwegian Aquaculture Register. To validate owner information, we used the Norwegian Aquaculture Register to verify that location numbers listed in VetReg corresponded to aquaculture sites producing juvenile salmonids.

To assess the validity of the ‘number of animals’ column, we used the number of doses reported within each record as a means of internal validation. As we compared based on the number of doses, this did not include bath vaccines that could not be converted from mL back to doses. For the injectable vaccines, the Summary of Product Characteristics (SPC) leaflets specified that every injectable vaccine here should be given as 1 dose per fish. In principle, the number of doses should therefore be equal to the number of animals. We used the information linked to the national product ID to calculate number of doses based (for records reported in “ml”, number of doses = quantity of vaccine in a dose * quantity reported, for records reported in “stk”, number of doses = quantity reported).

### Completeness

The ECDC (2014) defines completeness of reporting as the “absence of underreporting” [18]. We assessed external completeness by comparing the yearly number of fish vaccines reported in VetReg to wholesaler-based statistics of pharmaceutical sales obtained from the Norwegian Public Health Institute. We also refer to this as the coverage of VetReg. To perform the comparison, we converted the wholesales data to units, where $unit=pack\;size\; \times\; number\;of\;packs$ and we considered the wholesale numbers of packs sold as the reference standard. We calculated completeness in percent. Coverage below or above 100 percent, meant less use or more use compared to sales, respectively. To account for errors reported at the product ID level, we performed the comparison at both the product ID level and the vaccine name level.

### Timeliness

The timeliness of a register refers to the time between an event occurring and the registration of that event. To assess the timeliness of the register, we calculated the difference between the date of dispensing and the date of registration in VetReg. For 2016–2022, we graphed the timeliness as the percentage of new reports by the time since dispensing. We also investigated changes in timeliness from year to year.

### Descriptive analysis

We performed all analyses in R 4.4.0 and RStudio version 2023.09 [19, 20]. We used the following R packages: dplyr [21], lubridate [22], stringr [23], tidyr [24], ggplot2 [25], and reshape2 [26].

## Results

### Data description

For 2016–2022, there were 7,443 reports of fish vaccines in VetReg. The yearly number of reports during this period ranged from 715–1,283. We removed 12 entries that were recorded as “Notification of animal health personnel’s use of medicinal products.” There were 7,431 records remaining of fish vaccines dispensed by pharmacies. All of the reports to animal owners were submitted by four pharmacies.

### Validity

The dataset included 18 different fish vaccines with 23 unique national product IDs. Tables 1 and 2 describe the vaccines and their reporting to VetReg by national product ID. We added “Bath” at the end of the name for bath vaccines to differentiate those from injectable vaccines of the same name.

**Table 1:** Characteristics of fish vaccines reported in VetReg in 2016-2022

Product ID	ATC code	Vaccine name	Pack size	Dose	Type	Authorization years
480101	QI10AB04	Alpha Erm Salar Bath	1000 ml		Bath	2016 – Present
556139	QI10AB04	Alpha Erm Salar	250 ml	0.025 ml	Injection	2020 – Present
429437	QI10AB04	Alpha Erm Salar	500 ml	0.025 ml	Injection	2021 – Present
130772	QI10AB02	Alpha Ject 3000	500 ml	0.1 ml	Injection	2009 – Present
101148	QI10AB03	Alpha Ject 5–3	500 ml	0.1 ml	Injection	2011 – Present
101159	QI10AL02	Alpha Ject 6–2	500 ml	0.1 ml	Injection	2010 – Present
027475	QI10AL02	Alpha Ject Micro 6	1 × 500 ml	0.05 ml	Injection	2011 – Present
027464	QI10AL02	Alpha Ject Micro 6	1 × 250 ml	0.05 ml	Injection	2011 – Present
034501	QI10AA01	Alpha Ject micro 1 PD	500 ml	0.05 ml	Injection	2017 – Present
034490	QI10AA01	Alpha Ject micro 1 PD	250 ml	0.05 ml	Injection	2017 – Present
167812	QI10AB03	Alpha Ject micro 5	1 × 500 ml	0.05 ml	Injection	2022 – Present
465067	QI10AL04	Alpha Ject micro 7 ILA	500 ml	0.05 ml	Injection	2019 – Present
090235	QI10AL04	Alpha Ject micro 7 ILA	250 ml	0.05 ml	Injection	2019 – Present
560340	QI10AL02	Aquavac 6	500 ml	0.1 ml	Injection	2016 – 2023
189864	QI10AA01	Aquavac PD	500 ml	0.1 ml	Injection	2017 – 2021
193107	QI10AL05	Aquavac PD7	500 ml	0.1 ml	Injection	2015 – Present
61902	QI10BB03	Aquavac Relera Bath	1000 ml		Bath	2012 – 2023
472689	QI10AA02	Clynav	1 × 250 ml	0.05 ml	Injection	2018 – Present
169401	QI10BB02	Lipogen Duo	500 ml	0.1 ml	Injection	2006 – 2018
130420	QI10BB02	Lipogen Duo	1000 ml	0.1 ml	Injection	2006 – 2018
515591	QI10AA01	Norvax Compact PD	1 × 500 ml	0.1 ml	Injection	2012 – 2022
099126	QI10AL02	Norvax Minova 6	500 ml	0.1 ml	Injection	2010 – 2017
130519	QI10AL02	Pentium Forte Plus	500 ml	0.1 ml	Injection	2007 – Present

The table includes the national product ID, ATC code, vaccine name, pack size, dose, type, and years authorized in Norway of fish vaccines reported in VetReg in 2016–2022

**Table 2:** Overview of reporting in VetReg for fish vaccines in 2016–2022

Product ID	Vaccine name	No. records	Years	Units	Species
480101	Alpha Erm Salar	680	2016–2022	stk, ml, kg	Atlantic salmon, Rainbow trout, Brown trout
556139	Alpha Erm Salar	380	2020–2022	ml, stk	Atlantic salmon
429437	Alpha Erm Salar	187	2021–2022	ml, stk, g	Atlantic salmon
130772	Alpha Ject 3000	94	2016–2022	ml, stk	Turbot fish, Rainbow trout, Atlantic salmon, Brown trout
101148	Alpha Ject 5–3	267	2016–2022	stk, ml	Rainbow trout, Atlantic salmon, Brown trout, Ornamental fish, Cyclopterus
101159	Alpha Ject 6–2	249	2016–2022	stk, ml	Atlantic salmon, Rainbow trout, Brown trout, Other farmed fish, Ornamental fish
027475	Alpha Ject Micro 6	2,670	2016–2022	ml, stk, g, kg	Atlantic salmon, Brown trout, Ornamental fish, Bivalve molluscs, Rainbow trout
027464	Alpha Ject Micro 6	97	2016–2022	stk, ml	Atlantic salmon, Ornamental fish
034501	Alpha Ject micro 1 PD	860	2017–2022	ml, stk, kg	Atlantic salmon, Rainbow trout, Brown trout, Ornamental fish, Bivalve molluscs
034490	Alpha Ject micro 1 PD	43	2017–2019, 2021–2022	stk, ml	Atlantic salmon, Brown trout
167812	Alpha Ject micro 5	1	2022	stk	Atlantic salmon
465067	Alpha Ject micro 7 ILA	220	2020–2022	stk, ml	Atlantic salmon
090235	Alpha Ject micro 7 ILA	17	2020–2022	ml, stk	Atlantic salmon
560340	Aquavac 6	200	2016–2022	stk, ml	Atlantic salmon, Rainbow trout, Brown trout
189864	Aquavac PD	5	2018, 2020–2021	ml, stk	Atlantic salmon
193107	Aquavac PD7	464	2016–2021	ml, stk	Atlantic salmon, Brown trout, Bivalve molluscs
061902	Aquavac Relera	14	2016–2017, 2020–2022	stk, ml	Atlantic salmon, Rainbow trout, Brown trout
472689	Clynav	495	2020–2022	stk, ml	Atlantic salmon, Other farmed fish, Brown trout
169401	Lipogen Duo	13	2016–2017	ml	Atlantic salmon, Brown trout, Rainbow trout
130420	Lipogen Duo	1	2016	ml	Brown trout
515591	Norvax Compact PD	136	2016–2021	ml, stk, kg	Atlantic salmon
099126	Norvax Minova 6	1	2017	ml	Atlantic salmon
130519	Pentium Forte Plus	336	2016–2021	stk, ml	Atlantic salmon, Rainbow trout

The table includes the national product ID, vaccine name, number of records, years present in the registry, units and species reported in VetReg in 2016-2022

The pharmacies mainly reported usage in units of ‘stk’ (which is dose) or ‘ml’, but some entries were in ‘g’ and ‘kg’. For injectable vaccines reported in doses, we used information linked to the national product ID to convert doses to ml for 3,129 entries. For records reported in ‘g’ and ‘kg’, we manually corrected five entries and we excluded three entries in kg that could not be ascertained. The register included reports for two different bath vaccines, which are dissolved in water before administration. In 2016 and 2017, both types of bath vaccines contained entries with a mixture of reporting units in doses and ml, which were excluded from further analysis. In 2020–2022, the Aquavac Relera vet bath vaccine was reported exclusively in ‘ml’.

The VetReg data included information about the species, weight, animal owner, and number of animals. As expected, the most commonly reported species associated with the vaccines were Atlantic salmon, rainbow trout, and brown trout. In addition, some reports had other species listed and these could represent off-label usage; for animal species which have not been documented as target animals during registration of the vaccines. The listing of other species could also be caused by incorrect entries. All six entries of ornamental fish were investigated especially. These records were of vaccines dispensed for 500,000 to 1,285,714 fish, and these vaccines were therefore most likely for other, food-producing fish species.

The reported fish weights were between 0.001 and 1,685,000. However, we could not find a unit associated with this column. For records reported in 2020–2022, an animal owner was reported for 98 percent of the records. For salmonids, we assumed that the animal owners are hatchery locations, where the fish are vaccinated before being put to sea, identified by an official location number. We found that all of the records with reported animal owners matched to a hatchery location in the Norwegian aquaculture register.

We found that the number of animals reported was not consistent with the number of doses for 31 percent of the records investigated.

We tried to identify any systematic causes of the inconsistent records. We found that there were higher odds, odds ratio of 1.86 (95% CI 1.62–2.14, *p* < 0.001) of inconsistent reports when pharmacies reported multiple vaccines under the same report ID, meaning the same prescription. For example, pharmacies reported the same number of animals even when different quantities of doses for different vaccines were sold. In some other cases, pharmacies reported the number of animals as the sum of all the doses sold for the different vaccines under the same report ID. Supplementary Fig. 1 shows examples of these two issues.

An additional source of inconsistency between the number of doses and number of animals was related to probable errors in the reported units. We manually corrected 35 records where the number of animals exactly matched the number of doses calculated from quantity given in ml (doses incorrectly coded as ml) or the number of animals exactly matched the number of doses (ml incorrectly coded as doses). All 35 records were inspected to ensure that the exact match between the number of doses and the number of animals wasn’t because of inconsistencies mentioned in the previous paragraph but because of errors in the reported unit.

### Completeness

**Fig. 1:**
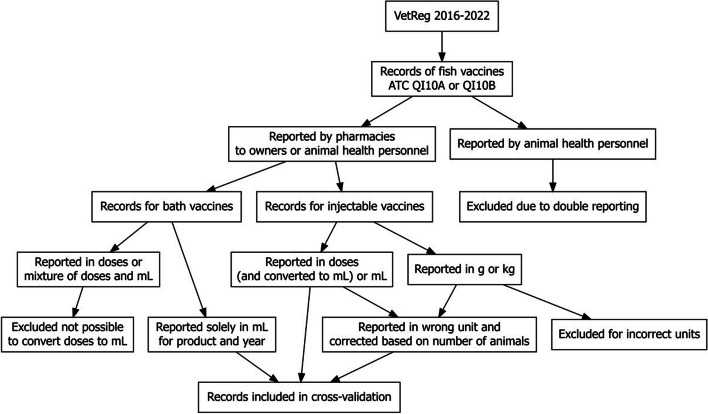
Diagram of records included and excluded from the completeness evaluation for fish vaccines reported in VetReg

To assess completeness, we compared the usage data in VetReg to wholesaler-based statistics at both at the national product ID level and per vaccine name for 2016–2022. Figure 1 and Table 3 give an overview of records included, excluded, or corrected for cross-validation. Due to reporting of quantity units in doses for bath vaccines, we removed 684 records, including those for the bath vaccine Alpha Erm Salar (national product ID: 480101) from all the years and all records for bath vaccine Aquavac Relera vet (national product ID: 061902) from years 2016 and 2017. Furthermore, due to invalid reporting of quantity units in ‘kg’ for injectable vaccines we removed three more entries from the cross-validation below. Therefore, the comparison was conducted on 6,744 entries (Table 3) from 22 product numbers (17 different vaccines).

**Table 3:** Data cleansing

Year	Total Records	Excluded records reported by vet	Excluded records for bath vaccines	Excluded records due to incorrect units	Reports with manually corrected units				Final records in completeness evaluation
					g to ml	kg to **L**	ml to stk	stk to ml	
2016	715	3	7 (0.98%)	0	0	0	5	0	705
2017	939	0	79 (8.41%)	0	0	0	9	0	860
2018	1060	1	144 (13.58%)	0	0	0	3	0	915
2019	1010	2	257 (25.45%)	0	1	3	10	1	751
2020	1172	0	114 (9.73%)	0	0	0	0	1	1058
2021	1283	2	33 (2.57%)	1	0	0	3	1	1247
2022	1264	4	50 (3.96%)	2	1	0	2	0	1208
Total	7443	12	684 (9.19%)	3	2	3	32	3	6744

The table gives an overview of the data cleansing process for data on fish vaccines reported in VetReg from 2016–2022, prior to completeness evaluation. It gives the number of records excluded due to double reporting by veterinarians in addition to pharmacies, because of units used when reporting bath vaccines that could not be compared to wholesaler data (and the percent of bath vaccine records excluded) and from reporting of an incorrect unit for use, which were not possible to correct. The table also provides the number of records where units were corrected prior to completeness evaluation

We first compared coverage at the product ID level. In general, we found that coverage varied substantially between products and years (SI Fig. 2, SI Table 1). The total coverage for different products ranged from 35–283 percent. There were five vaccines with multiple product numbers due to them being sold in various pack sizes (Alpha Ject Micro 6, Alpha Ject micro 1 PD, Alpha ERM Salar, Alpha Ject micro 7 ILA, Lipogen Duo). We observed that within the same vaccines, individual product IDs were subject to under and over reporting. In other words, if a vaccine was sold in two pack sizes; the use of one of them could be over reported compared to sales data while the other was under reported.

**Fig. 2:**
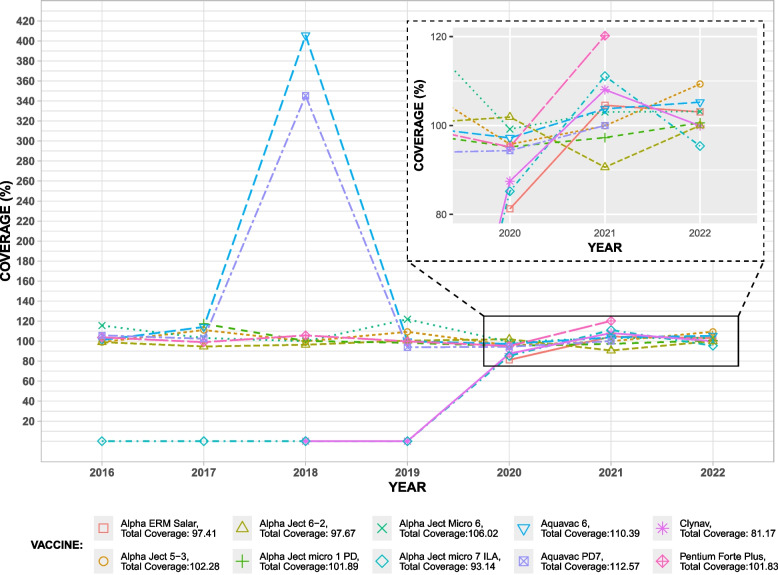
The figure shows yearly and total coverage for the ten most used fish vaccines, excluding bath vaccines, reported to VetReg in 2016–2022 as compared to wholesales statistics. Vaccines product IDs are shown in parentheses, those with multiple products are aggregated under a single vaccine name

We repeated our analysis at the vaccine name level. Figure 2 and Table 4 provide an overview of total and yearly coverage for each vaccine. We found that combining product numbers improved the overall coverage of the vaccines with multiple product numbers. For example, Alpha Ject Micro 1 PD had product level coverage of 103 percent and 65 percent, but a combined coverage of 102 percent. In general, VetReg had good coverage for most of the vaccines at vaccine name level as compared to the wholesaler statistics. However, this was not the case for all vaccines over the entire study period. For example, Clynav vet inj and Alpha Ject Micro 7 ILA, were not reported in VetReg prior to 2020, even though these appear in wholesaler data prior to 2020. At the same time, Norvax Minova 6 vet inj, appeared in VetReg in 2017 but not the wholesaler data for any year. However, this vaccine was used in very small quantity and accounted for only one record.

**Table 4:** Yearly and total coverage

Name	Product ID	Yearly coverage (%)							Total
		2016	2017	2018	2019	2020	2021	2022	
Alpha Ject Micro 6	027464, 027475	115.54	103.07	99.92	121.8	99.18	103.04	103.14	106.02
Pentium Forte Plus	130519	103.72	98.95	105.73	99.71	95.17	120.19	-	101.83
Alpha Ject micro 1 PD	034490, 034501	-	117.26	100.19	98.23	95.18	97.28	100.61	101.89
Clynav	472689	-	-	0	0	87.48	108.05	99.92	81.17
Aquavac PD7	193107	105.75	102.33	345.22	93.86	94.35	100	-	112.57
Alpha Ject 5–3	101148	99.02	111.06	99.85	109.28	95.77	99.92	109.32	102.28
Alpha Ject 6–2	101159	99	94.61	96.4	100.45	101.89	90.61	100	97.67
Alpha ERM Salar	429437, 556139	-	-	-	-	81.25	104.54	103.05	97.41
Alpha Ject micro 7 ILA	465067, 640117, 090235	0	0	0	0	85.19	111.1	95.38	93.14
Aquavac 6	560340	101.07	114.13	405.61	99.74	97.2	103.75	105.25	110.39
Norvax Compact PD	515591	98.94	106.1	145.78	99.26	73.16	100	-	102.07
Alpha Ject 3000	130772	223.57	129.82	106.25	96.16	121.02	124.46	105.15	116.8
Aquavac Relera Bath	061902	Excluded	Excluded	-	-	85.93	100.00	100.00	Excluded
Lipogen Duo	169401, 130420	99.75	64.5	-	-	-	-	-	81.25
Pentium Forte Plus ILA	1102444	0	-	-	-	-	-	-	0
Aquavac PD	189864	-	-	42.39	-	Not in sales	Not in sales	-	283.15
Alpha Ject micro 5	167812	-	-	-	-	-	-	100	100
Norvax Minova 6	099126	-	Not in sales	-	-	-	-	-	Not in sales

The table gives a summary of coverage (in percentage) for fish vaccines reported in VetReg for 2016–2022 as compared to wholesaler statistics. Dashes indicate that no data was reported in either VetReg or wholesaler data, ‘Not in sales’ indicates that usage was reported in VetReg but not in wholesaler data, ‘Excluded’ is used for bath vaccines reported to VetReg in mL that cannot be converted to doses

### Timeliness

**Fig. 3:**
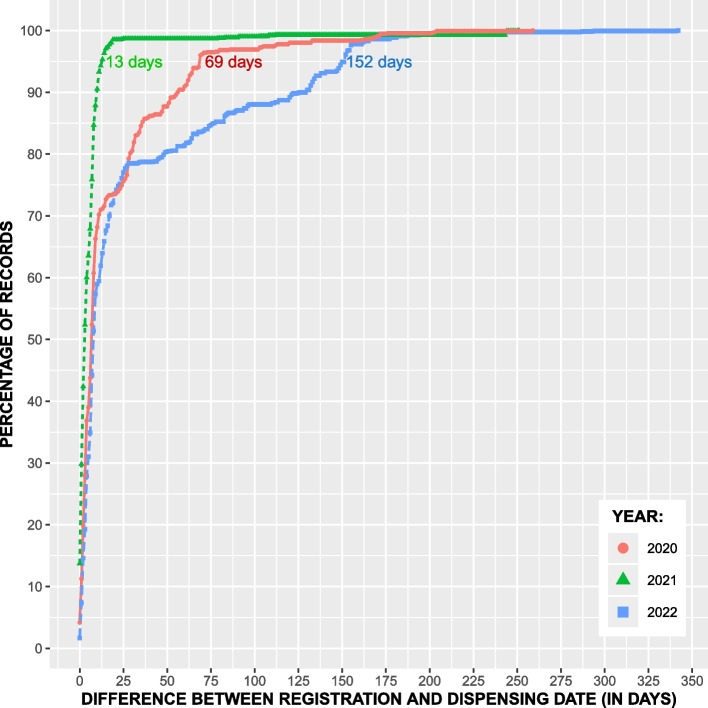
The figure shows the timeliness of reports to VetReg for fish vaccination data for 2020–2022. The number on each curve marks the point when 95 percent of records are received in a given year. The percentage of records was calculated by dividing the number of records with a difference between registration and dispensing date less than or equal to days on y-axis by the total number of records registered in a particular year

For vaccine reports, the time it took for 95 percent of the records to be submitted ranged from 14–152 days per year in 2016–2022 (SI Table 2) and 13–152 days for 2020–2022 (Fig. 3). We observed that the timeliness was sporadic between years, with no trend. Over the study period, 99.28% of all records were reported by two out of the four pharmacies, with pharmacy A reporting 53.16% of the records and pharmacy B reporting 46.12% of the records. In terms of timeliness, Pharmacy A took 47 days on average to report 95 percent of the records whereas pharmacy B took 13 days on average to do the same. However, for all years, 75 percent of the reports were submitted in 25 days or less.

Table 5 summarizes all identified quality issues.

**Table 5:** Summarized data quality issues

Attributes	Variable names	Issues identified
Validity	Quantity unit	Wrong unit-uncorrectable
		Wrong unit-correctable
	Number of animals	Wrong number reported
	Species of fish	Wrong species reported
	National product ID	Wrong pack size reported
Completeness		Variable completeness between vaccines
		Variable completeness over years
		Non reported use: specific vaccines for some years
Timeliness		Long time to reach 95 percent reporting

The table shows a summary of the quality issues identified in fish vaccine data in VetReg 2016–2022. Three different quality attributes were evaluated and for validity, four variables from VetReg were investigated

## Discussion

Overall, the completeness of VetReg for injectable fish vaccines was adequate compared to sales data for 2020–2022. In these years, coverage in VetReg for different vaccines was 73 to 124 percent of the corresponding sales per year. The coverage was more consistent for the top ten vaccines sold in 2020–2022, ranging from 81 to 113 percent. We found that 2022 had the most complete records, with between 95 and 109 percent coverage for all vaccines. There was a marked increase in completeness after 2020, as compared to earlier years in the study (2016–2019). To our knowledge, there were no major changes in the aquaculture industry in Norway, the distribution of medicines or the registries during this time, which could explain this change. Due to the reporting of bath vaccines in doses, we could not evaluate the coverage of bath vaccines reported in VetReg to the wholesales data for all vaccines in all years. This is because it is not possible to convert dose units to mL for these vaccines, since the number of fish that can be vaccinated using the same volume of bath vaccine can vary. However, the completeness for one bath vaccine reported only in mL for 2020–2022 was 85–100 percent, within the range for the injectable vaccines.

When comparing VetReg to wholesales data, we can expect some over- and under- reporting. This is due to fundamental differences between the two registers. Reports in VetReg include a date of delivery to a customer. By contrast, if wholesalers report through their accounting system and if there is a lag between when the medicines are delivered to customers and when they are billed, medicines can be delivered one year and reported sold the next. Although, we do not expect these differences to be large. We did find large differences between the two registers for some of the vaccines, particularly in 2016–2019. For example, two vaccines, Cylnav and Alpha Ject micro 7 ILA, were not in VetReg prior to 2020, despite having marketing authorization from 2018 and 2019, respectively. In the first years of authorization, they were only reported in wholesalers data and not in VetReg. Autogenous vaccines were only found in the wholesaler data. We do not know the exact reason for these discrepancies in VetReg, but we can speculate that they arise from a systematic failure to register certain vaccines. In other cases, we found that the incorrect registration of quantity units also reduced the calculated coverage, since not all units were possible to convert to the number of packages. We also found four examples of vaccines reported used one year, but not sold by wholesalers the same year or the year before. There were several examples of more than 100 percent coverage of VetReg data compared to wholesalers data. If the use is correctly reported, this indicates that wholesaler data might also not be fully complete, meaning that not all wholesalers report all sales.

In this study, we assessed the validity of individual variables within VetReg. The data mainly contained two units for amounts sold (milliliter and doses) and we used vaccine-specific conversion factors (dose in ml) to summarize most VetReg-entries per package ID and vaccine name. We found that coverage improved when comparing data per vaccine name instead of per package ID, indicating that some reports had incorrect package IDs, but correct vaccine names. This inaccuracy in reporting the use of the actual package size to VetReg did not affect the possibility of calculating the number of doses used of a particular vaccine.

Within individual records, the number of animals vaccinated was not always equal to the number of doses sold. This occurred especially when multiple vaccines were issued on the same prescription. However, we could use the ‘number of animals’ variable to identify records with incorrect quantity units in cases where only one vaccine was issued per prescription. The potential use for correctly reported number of animals would have been internal validation of the reported amounts used.

The need to convert, exclude, or correct quantity units to use the register highlights the importance of harmonizing how vaccine usage is reported. By comparison, all quantities are reported in packages from pharmacies in VetReg for the sale of antibiotics [27]. The ‘weight’ variable (describing weight of fish) was also problematic, since no specified units for weights rendered this information unusable.

For timeliness, we found that the mean time to registration was not consistent between years, and there was no trend for improved timeliness in more recent years. Moreover, we could not find a legal deadline for when pharmacies have to report to VetReg. This implies that users of data from VetReg cannot know when to expect all records for a given period to be entered into VetReg. In our study period, it took up to 154 days to get 95 percent of a year’s records in VetReg. Since we received 2022 VetReg data in 2024, we regard that the conclusions we draw from 2022-data are not affected by the timeliness.

Previous studies have assessed the quality of VetReg for different purposes. Remen and Sætre (2018) investigated the quality of data on antiparasitics used against salmon lice in VetReg [14]. They discovered issues with the validity of the amounts reported, for example, they found instances where the number of fish were reported as the amount of product [14]. As with our study, they also found that data quality improved over the years [14]. Grave et al. [13], who investigated the quality of antibiotics reporting in VetReg, found better coverage of VetReg data compared to sales data, for antibiotics prescribed to fish compared to antibiotics prescribed or used for land animals. Grave and Hopp [27] also performed a quality assessment of VetReg data on antibiotics. They discovered two main quality issues, the validity of amounts reported from pharmacies (decimal numbers were not received by the system and only whole numbers were found in VetReg) and issues with units used for reporting by veterinarians. These units did not always match the strength unit for the same product, thereby making it difficult to calculate amounts used. This exact issue is not relevant for vaccines as the strength units for vaccines are not relevant for reporting, e.g. as the strength of a vaccine may relate to the serological response in the fish. Together with the findings of our study, this emphasizes the need to perform quality assessments of VetReg data per product category and to take into account the data provider. Quality should also be investigated on data from the entire relevant period, as it has been shown to change over time.

In our opinion, VetReg data from 2020 are of sufficient quality to present the vaccine use on a national level, with the exception of the bath vaccines. From the VetReg data, it is possible to evaluate if the hatcheries used only legal vaccines, as long as all vaccines are bought from pharmacies. We believe this to be the case in Norway as the product the hatcheries are selling is smolt vaccinated with given vaccines. It is however not possible to evaluate which autogenous vaccines are dispensed by the pharmacies, because sales of such vaccines are only present in wholesaler data. Since these vaccines are not included in the national product registry, the metadata on them, such as active substances, dose and ATC-code, are not available. Autogenous vaccine data are difficult to find in a register and, if found, difficult to evaluate the quality of. We have only evaluated the coverage of vaccines found in VetReg and the autogenous vaccines are therefore not included.

Given the assumption that all fish are vaccinated with the vaccines NFSA considers irresponsible not to use, doses of those vaccines can be used as a denominator in calculating the proportion of fish vaccinated with various types of vaccines at a national, regional and hatchery level. The regional level is only valid for where the hatcheries are located, because fish might be put to sea in another region than where the hatchery is located. Our inability to convert bath vaccines from doses to ml and vice versa creates a challenge for reporting usage of these products, which has been highlighted in a previous study by Bravo and Midtlyng [28] on the vaccine usage in the Chilean aquaculture industry [28]. The inaccuracy of the ‘number of animals’ column in VetReg means that there is no other source of information for the number of fish vaccinated with bath vaccines. In order to be able to evaluate completeness of bath vaccines and report the number of fish vaccinated with this subcategory of vaccines, the unit for amount must be ml and number of fish must be reported accurately. The fact that similar quality issues were identified in a Chilean study, points to the general importance of quality evaluation of health registry data, prior to further use of the data.

There are no publically available data on the vaccine status on a sea farm level and it is not possible to trace which hatchery(ies) the fish in a farm originate from using register data. This means that some of the questions regarding vaccine side-effects, efficacy and the effects of vaccines on the spread and severity of disease outbreaks cannot be investigated on a farm-level using VetReg as a source of vaccine status. Therefore, farm-level vaccine status must be obtained through, for example, questionnaires or access to farm records, as was done in the study by Jensen et al. [9].

## Conclusions

The completeness of the data in VetReg for injection vaccines for Atlantic salmon and rainbow trout were considered to be adequate for the period 2020 to 2022, when evaluated per vaccine name. The validity of the units used for reporting had some quality issues, especially for bath vaccines. A uniform use of units (ml) will improve the quality. The validity issue of the ‘number of animals’ variable was associated with prescriptions with multiple vaccines, and this variable should be correctly reported per vaccine. Since few pharmacies dispense fish vaccines, quality improvement could be achieved with targeted information for each pharmacy. Almost all records included an owner location, which means that VetReg records can be used to present proportions of fish vaccinated at regional or hatchery level. Increased timeliness in fish vaccine records will expand the possible use of the most current records, including real-time or near real-time monitoring of use. Setting a deadline for reporting could increase timeliness. VetReg, alone or in combination with other publically collected records, are currently not suited to describe vaccine status on a sea farm level.

## Supplementary Information

Supplementary Material 1. Supplementary Fig. 1 Number of animals examples. This figure shows examples of reports in VetReg with the same report ID, showing (a) pharmacies sometimes reporting the same number of animals even when different quantities of doses for different vaccines were sold, (b) pharmacies reporting the number of animals as the sum of all the doses sold for the different vaccines under the same report ID. The report IDs have been anonymized and the names of the column names changed from Norwegian to English.

Supplementary Material 2. Supplementary Fig. 2 Yearly and total coverage for the ten most used fish vaccines. This figure shows yearly and total coverage for the ten most used fish vaccines by product ID, excluding bath vaccines, reported to VetReg in 2016–2022 as compared to wholesales statistics.

Supplementary Material 3. Supplementary Table 1 Coverage at the product level. The table includes a summary of coverage (in percentage) for fish vaccines at the product-level reported in VetReg for 2016–2022 as compared to wholesaler statistics. Dashes indicate that no data was reported in either VetReg or wholesaler data and ‘Not in sales’ indicates that usage was reported in VetReg but not in wholesaler data.

Supplementary Material 4. Supplementary Table 2 Timeliness. Timeliness of reports to VetReg for fish vaccination data for 2016–2022, reported as number of days before 50, 75 and 95 percent of the records.

## Data Availability

The data that support the findings of this study are available from the Norwegian Food Safety Authority (VetReg) and the Norwegian Institute of Public Health (wholesaler data) but restrictions apply to the availability of these data, which were used under license for the current study, and so are not publicly available. Data can however be requested from the same data providers by others.

## Abbreviations

ATC: Anatomical Therapeutic Chemical
NIPH: Norwegian Institute of Public Health
NFSA: Norwegian Food Safety Authority
NoMA: Norwegian Medicinal Products Agency
SPC: Summary of Product Characteristics
VetReg: Norwegian Veterinary Prescription Register
