# Supplementary figures and images for: Quality assessment of fish vaccine data in the Norwegian Veterinary Prescription Register (VetReg)
Source: BMC Vet Res. 2025 Jan 13;21:17. doi: 10.1186/s12917-024-04460-7 (PMC11727185; doi:10.1186/s12917-024-04460-7)

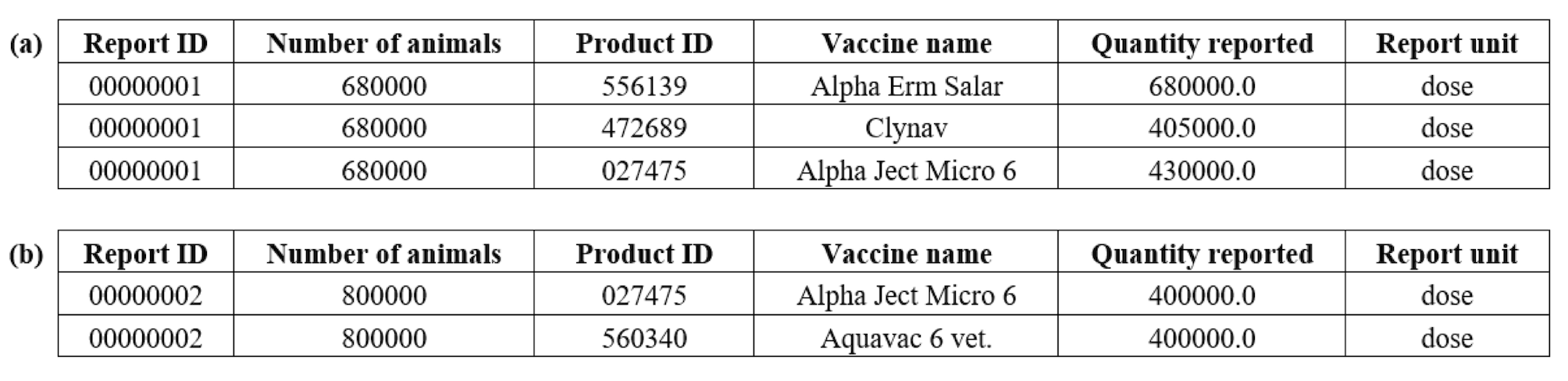

Supplement: Supplementary file 1 — Supplementary Material 1. Supplementary Fig. 1 Number of animals examples. This figure shows examples of reports in VetReg with the same report ID, showing (a) pharmacies sometimes reporting the same number of animals even when different quantities of doses for different vaccines were sold, (b) pharmacies reporting the number of animals as the sum of all the doses sold for the different vaccines under the same report ID. The report IDs have been anonymized and the names of the column names changed from Norwegian to English. [file 12917_2024_4460_MOESM1_ESM.tiff]

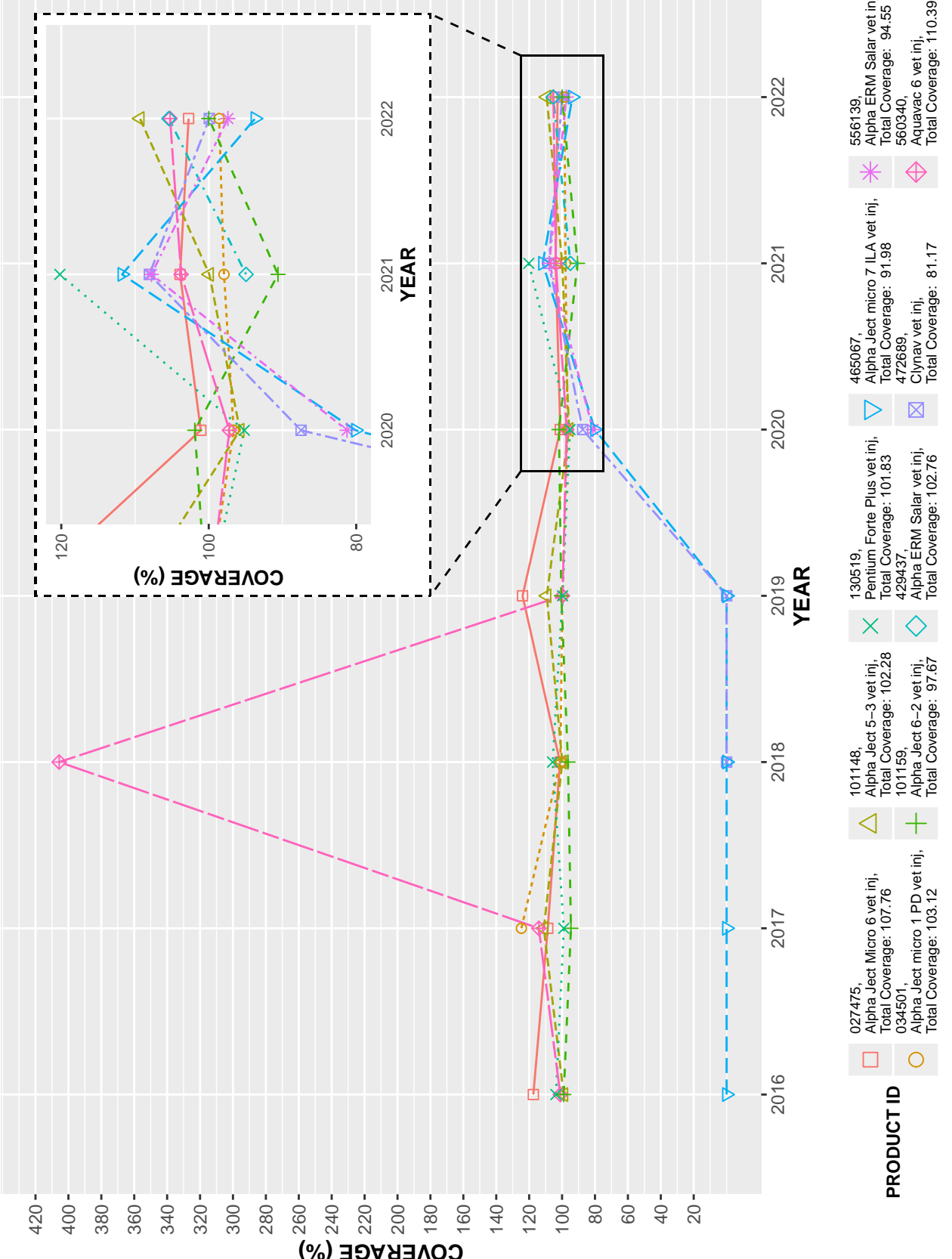

Supplement: Supplementary file 2 — Supplementary Material 2. Supplementary Fig. 2 Yearly and total coverage for the ten most used fish vaccines. This figure shows yearly and total coverage for the ten most used fish vaccines by product ID, excluding bath vaccines, reported to VetReg in 2016–2022 as compared to wholesales statistics. [file 12917_2024_4460_MOESM2_ESM.pdf]
