# Supplementary material for: Quality assessment of fish vaccine data in the Norwegian Veterinary Prescription Register (VetReg)
Source: BMC Vet Res. 2025 Jan 13;21:17. doi: 10.1186/s12917-024-04460-7 (PMC11727185; doi:10.1186/s12917-024-04460-7)
Supplement: Supplementary file 3 — Supplementary Material 3. Supplementary Table 1 Coverage at the product level. The table includes a summary of coverage (in percentage) for fish vaccines at the product-level reported in VetReg for 2016–2022 as compared to wholesaler statistics. Dashes indicate that no data was reported in either VetReg or wholesaler data and ‘Not in sales’ indicates that usage was reported in VetReg but not in wholesaler data. [file 12917_2024_4460_MOESM3_ESM.docx]

Supplementary Table 1 Coverage at the product level

| **Product ID** | **Name** | **Yearly coverage (%)** | | | | | | | **Total** |
| --- | --- | --- | --- | --- | --- | --- | --- | --- | --- |
|  |  | **2016** | **2017** | **2018** | **2019** | **2020** | **2021** | **2022** |  |
| 556139 | Alpha ERM Salar | - | - | - | - | 81.25 | 107.78 | 97.41 | 94.55 |
| 429437 | Alpha ERM Salar | - | - | - | - | - | 94.94 | 105.40 | 102.76 |
| 130772 | Alpha Ject 3000 | 223.57 | 129.82 | 106.25 | 96.16 | 121.02 | 124.46 | 105.15 | 116.80 |
| 101148 | Alpha Ject 5-3 | 99.02 | 111.06 | 99.85 | 109.28 | 95.77 | 99.92 | 109.32 | 102.28 |
| 101159 | Alpha Ject 6-2 | 99.00 | 94.61 | 96.40 | 100.45 | 101.89 | 90.61 | 100.00 | 97.67 |
| 027475 | Alpha Ject Micro 6 | 117.45 | 108.70 | 101.25 | 124.01 | 101.08 | 103.89 | 102.73 | 107.76 |
| 027464 | Alpha Ject Micro 6 | 54.28 | 0.40 | 7.40 | 62.99 | 9.55 | 31.00 | 145.25 | 35.15 |
| 034501 | Alpha Ject micro 1 PD | - | 124.71 | 101.02 | 99.85 | 96.62 | 97.93 | 98.59 | 103.12 |
| 034490 | Alpha Ject micro 1 PD | - | 41.37 | 74.23 | 6.61 | 0 | 45.27 | 205.88 | 64.89 |
| 167812 | Alpha Ject micro 5 | - | - | - | - | - | - | 100.00 | 100.00 |
| 640117 | Alpha Ject micro 7 ILA | - | - | - | 0 | - | - | - | 0 |
| 465067 | Alpha Ject micro 7 ILA | 0 | 0 | 0 | 0 | 80.08 | 111.75 | 93.71 | 91.98 |
| 090235 | Alpha Ject micro 7 ILA | - | 0 | - | - | Not in sales | 34.17 | 240.99 | 222.04 |
| 560340 | Aquavac 6 | 101.07 | 114.13 | 405.61 | 99.74 | 97.20 | 103.75 | 105.25 | 110.39 |
| 189864 | Aquavac PD | - | - | 42.39 | - | Not in sales | Not in sales | - | 283.15 |
| 193107 | Aquavac PD7 | 105.75 | 102.33 | 345.22 | 93.86 | 94.35 | 100.00 | - | 112.57 |
| 472689 | Clynav | - | - | 0 | 0 | 87.48 | 108.05 | 99.92 | 81.17 |
| 169401 | Lipogen Duo | 91.14 | 64.50 | - | - | - | - | - | 77.16 |
| 130420 | Lipogen Duo | Not in sales | - | - | - | - | - | - | Not in sales |
| 515591 | Norvax Compact PD | 98.94 | 106.10 | 145.78 | 99.26 | 73.16 | 100.00 | - | 102.07 |
| 099126 | Norvax Minova 6 | - | Not in sales | - | - | - | - | - | Not in sales |
| 130519 | Pentium Forte Plus | 103.72 | 98.95 | 105.73 | 99.71 | 95.17 | 120.19 | - | 101.83 |
| 1102444 | Pentium Forte Plus ILA | 0 | - | - | - | - | - | - | 0 |

The table includes a summary of coverage (in percentage) for fish vaccines at the product-level reported in VetReg for 2016-2022 as compared to wholesaler statistics. Dashes indicate that no data was reported in either VetReg or wholesaler data and ‘Not in sales’ indicates that usage was reported in VetReg but not in wholesaler data.
