# Supplementary material for: Quality assessment of fish vaccine data in the Norwegian Veterinary Prescription Register (VetReg)
Source: BMC Vet Res. 2025 Jan 13;21:17. doi: 10.1186/s12917-024-04460-7 (PMC11727185; doi:10.1186/s12917-024-04460-7)
Supplement: Supplementary file 4 — Supplementary Material 4. Supplementary Table 2 Timeliness. Timeliness of reports to VetReg for fish vaccination data for 2016–2022, reported as number of days before 50, 75 and 95 percent of the records. [file 12917_2024_4460_MOESM4_ESM.docx]

Supplementary Table 2 Timeliness

| **Year** | **Difference between registration date and dispensing date (in days):** | | |
| --- | --- | --- | --- |
|  | **95 percent of records** | **75 percent of records** | **50 percent of records** |
| 2016 | 14 | 8 | 6 |
| 2017 | 30 | 14 | 8 |
| 2018 | 25 | 9 | 4 |
| 2019 | 9 | 6 | 2 |
| 2020 | 69 | 25 | 7 |
| 2021 | 13 | 7 | 3 |
| 2022 | 152 | 23 | 8 |

Timeliness of reports to VetReg for fish vaccination data for 2016-2022, reported as number of days before 50, 75 and 95 percent of the records.
